# Past, Present and Future: Combining habitat suitability and future landcover simulation for long-term conservation management of Indian rhino

**DOI:** 10.1038/s41598-020-57547-0

**Published:** 2020-01-17

**Authors:** Tanoy Mukherjee, Lalit Kumar Sharma, Goutam K. Saha, Mukesh Thakur, Kailash Chandra

**Affiliations:** 1 Zoological Survey of India, Prani Vigyan Bhawan, New Alipore, Kolkata, West Bengal India; 2 Department of Zoology, University of Calcutta, Kolkata, West Bengal India

**Keywords:** Ecology, Zoology

## Abstract

The Indian rhino (*Rhinoceros unicornis*) is susceptible to habitat change and fragmentation due to illegal logging, rapid urbanization and non-forest use and therefore were confined in to isolated areas throughout its distribution. The present study was conducted in Gorumara landscape which is composed of two protected areas (PAs) *viz*., Gorumara National Park (GNP) and Chapramari Wildlife Sanctuary. Both PAs were separated by a territorial forest range (Bridge Area), which is between both the PAs and under high anthropogenic disturbance. The study was designed to understand the impacts of landcover change on habitat suitability of *R*. *unicornis* in a multi-temporal scenario from 1998 to 2018 using ensemble approach and also to simulate the future habitat suitability for 2028. Our result suggests a significant increase in woodland cover inside the PAs, whereas the grassland cover has increased outside the PA in territorial range. We found a strong positive association of *R*. *unicornis* with grasslands. The Comparison of the future suitability model of 2028 with that of 2018 indicates a substantial increase in rhino suitable area by 13% in the territorial forest. Hence, bringing the territorial forest into the PA network, will be a crucial step to increase the fodder availability and better connectivity for the long term survival of the species.

## Introduction

The Indian rhino (*Rhinoceros unicornis*) is one of the threatened mega-herbivores distributed in isolated Protected Areas (PAs) in the Northern foothills of India and Southern areas of Nepal. This species is a classic example of a conservation success story in Asia, where the population of the species has recovered from fewer than 200 individuals to about 3,557 individuals in India and Nepal, because of consented efforts of the forest management of both the governments^1^. Despite the efforts made by the forest managers towards its conservation, the species is still facing several conservation threats largely due to habitat loss in terms of fragmentation and encroachment^2–6^. The International Union for Conservation of Nature and Natural Resources (IUCN) has listed this species as vulnerable^7^. The Convention on International Trade in Endangered Species of Wild Fauna and Flora has also listed the species in Appendix-I since 1975 to curb down the illegal poaching of the species for trade. Unlike other widespread mega-herbivores, such as Asian elephant (*Elephas maximus*) the current rhino’s population are confined mainly in and around a few patches^7^. From the end of 15^th^-century rhinos were abundant in the floodplains of Ganges, Bramhaputra and Sindh rivers, with a distribution ranging from Indo- Burmese territories in the east to Pakistan in the west^8–10^. In India, the state of Assam constitutes the major stronghold with an estimated 2,625 individuals in four populations^1^, followed by Northern plains of Wes Bengal state with a total count of 280 individuals. These isolated populations are at significant risk of local extirpation due to increasing land cover change, which may result in loss of genetic connectivity, inbreeding and population bottlenecking. Moreover, the growth of population in fragmented patches along with other sympatric species may also lead to increases in competition for space and food, leading to violent intraspecific and interspecific competitions^11^.

The *R*. *unicornis* prefer grassland habitats intermixed with wetlands and riverine forest but with marked seasonal variations that are evident in their habitat utilisation patterns^9,12^. However, the dominance of tall grasses was well known to influence the *R*. *unicornis* distribution^13^ positively. These habitats are increasingly getting threatened due to riverside erosion, accumulation of weeds in water bodies such as water hyacinth and invasion by alien species such as *Mikania sp*. and *Albezzia procera* in grassland habitat^2–5^. In some of the populations of *R*. *unicornis*, the degradation of habitat in the form of loss of food resources is leading to crop damage in fringe villages which results in the development of antagonistic behaviour among the local communities towards the species^6^. In West Bengal state habitat fragmentation has resulted in the confinement of the species into two isolated areas namely Jaldapara National Park (JNP) with 204 individuals and Gorumara National Park (GNP) with ~50 individuals. Previous studies from the landscape attributed *R*. *unicornis* habitat loss to agriculture expansion, extension of tea gardens, encroachment, river erosion and improper forestry practices^14^. The *R*. *unicornis* habitat in greater Gorumara landscape is patchy, and is a mix of territorial forest ranges including Jalpaiguri, Kalimpong and Cooch Behar Forest Divisions and PAs such as Jaldapara Wildlife Sanctuary, Chapramari Wildlife Sanctuary (CWLS) and GNP^15^. The increasing patchy distribution of rhino habitat may lead to loss of movement corridors and further genetic stress^16–18^.

The study landscape is a known tourist destination in the region, and because of this tourist influx, the landscape is experiencing a significant amount of anthropogenic pressure, mostly because of the busy Railway line and vehicular traffic on the NH 31. The GNP and CWLS are two PAs which are separated by a railway line and national highways which are posing severe threats to the large body animals such as rhinos, elephants and gaur. Over the recent past, several individuals of these mega-herbivores got killed because of accidents^19^. The primary factor behind the accidents is the availability of seasonal habitat in the territorial forest areas of these mega-herbivores which motivates the animal to negotiate these dangerous linear features. The mega-herbivores, including *R*. *unicornis*, in particular, tend to track food resources distributed in the landscape^20^. The previous study has documented the movement of *R*. *unicornis* from PA into the agricultural landscape, which increases the likelihood of being killed in retaliation to crop damage by local communities and sometimes poached^21^.

The PA management strategy in the study landscape is mainly protection based, which includes fencing, heavy patrolling and monitoring. However, the focus has been on habitat-based management, which may not be suitable for the long term viability of large animals such as *R*. *unicornis*. The forested areas in the landscape are managed by following two different strategies, i.e.; the PAs are administered through action plans whereas, the non-PAs or territorial forests are managed under working plans. In India, PAs are kept aside for the conservation of species and its habitat, whereas the territorial forests are managed for production forestry as per the Indian Forest Act, (1972). This difference in management objectives may not always be useful for the long term viability of large size animals. Hence, it is imperative to adopt a landscape-based management strategy by focussing on the flagship species of the landscapes^22,23^. It is crucial to monitor the landcover configuration change for prioritisation of conservation and management strategies at local scales^24,25^. Moreover, the focus should be on the role of different degradation drivers on the ecological intactness, which is vital for the conservation of species^26^.

During the recent past, improvements in using remote sensing technology for monitoring change in the landscape had made a significant contribution in taking vital management decisions for conservation and management^27–29^. The remote sensing and GIS techniques provide necessary spatiotemporal support, which is relevant, reliable, quantifiable, and timely to cater information needed for making informed decisions^30,31^.

Recent development in remote sensing technologies have equipped researchers to develop models which are useful in monitoring and predicting the futuristic changes in landcover, and also imperative in making data driven policies^32–35^. These models are efficient in detecting the change in the landscape configuration by using quantifiable drivers such as historical land cover trends, topology and anthropogenic changes. Hence, large number of researchers have used these models with varied applications and dimensions^36–40^. However, recent studies have brought out the need of new technique which have better capabilities in quantifying the effects of drivers on the predictions^41^. Among the various existing models the cellular automata (CA) is the most widely used, which generates rich patterns and able to represent effectively the spatially nonlinear stochastic landcover change processes^42,43^. The Logistic Regression (LR) based models were generally used to predict the probability of occurrence of a particular event by the values of a set of features whereas, the machine learning such as Artificial Neural Network (ANN) uses backpropagation gradient calculation method which updates the weights of a multilayer perceptron^44,45^. Such strategy has been adopted by a number of researchers and found useful in predicting the future land cover^46,47^.

This study has been designed to assess land cover change in the study landscape, followed by habitat suitability analysis in the multi-temporal scenario by adopting the ensemble approach. The study provides two-decades of landscape change data to assess changes in the habitat suitability for *R*. *unicornis* across the study landscape. Further, we adopted both ANN and LR based strategies for predicting the future land cover and also to identify suitable habitats of *R*. *unicornis* for the year 2028.

## Results

### Land cover indicators and transformation dynamics from the year 1998 to 2018

**Figure 1:**
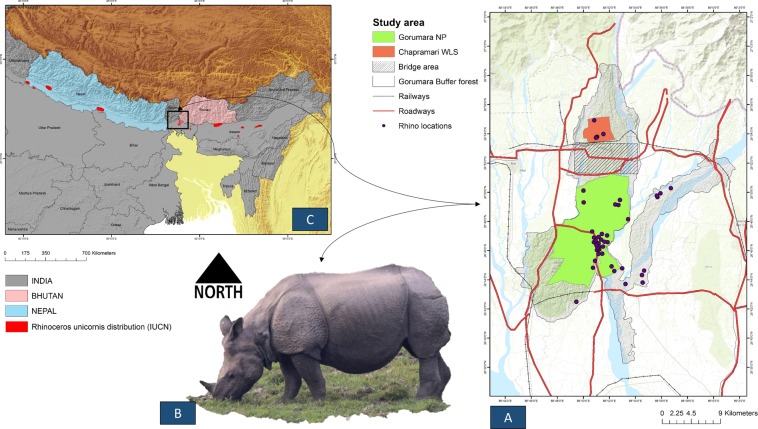
Showing study area map. (**A**) Showing Gorumara National Park (GNP), Chapramari Wildlife Sanctuary (CWLS) and Bridge area along with road and rail network in red and black lines respectively. Background topographic surface has been created by adding ESRI base map (Topographic) in ArcGIS 10.6. (**B**) Field picture of one-horned rhino (*Rhinoceros unicornis)* in its natural habitat of GNP. (**C**) The extant distribution of one-horned rhino (*Rhinoceros unicornis)* as per IUCN. (Maps are generated using ArcGIS 10.6: www.esri.com).

**Figure 2:**
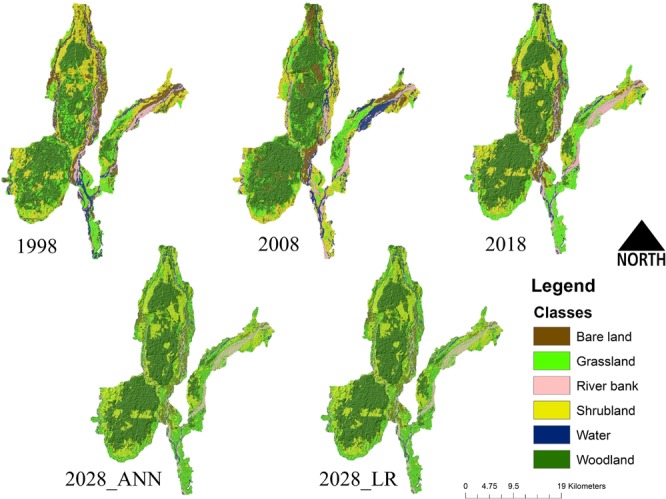
Representing the different landcover classes in GNP buffer forest, in multitemporal datasets. Upper panel starting from the left describes the landcover types in the year 1998, 2008 and 2018. The lower panel represents the stimulated ANN (Artificial neural network) and LR (Logistic Regression) models for the year 2028. (Maps are generated using ArcGIS 10.6: www.esri.com).

The supervised classification of the satellite images resulted in the identification of six dominant land cover types of the study area (Figs. 1, 2). The classification accuracy was assessed using 360 sample points (60 per class) generated using the stratified random sampling method over the study area for the LULC maps of 2018. The overall accuracy for 2018 map was 88.05%, and the kappa coefficient was 0.857, indicating a high degree of accuracy (Table. S1).

The comparisons between the multiple-temporal datasets (1998–2018) revealed that the grassland cover decreased initially in the first decade, followed by a sharp increase in the latter half (Figs. S1, S2). However, the shrubland cover has decreased continuously from 1998 to 2018 in two decades, and similar trends were also observed in case of bare land cover. A notable increase was found in the woodland cover, and it has increased from 101.61 Km^2^ of 1998 to 122.34 Km^2^ in 2018 (Figs. S1, S2).

The transition probability matrix indicated the highest probability of conversion from bare land to shrubland by a value of 0.5, followed by 0.47 and 0.34 for river bank to water and grassland to shrubland respectively between 1998 and 2008. Whereas, from 2008 to 2018, the probability of transition value (0.56) was highest from bare land to shrubland, followed by shrubland to grassland (0.51) (Table. S2).

### Simulated future land cover for the year 2028

The percentage correctness of the ANN-based future land cover was found to be 82.10%, with kappa statistical value of 0.756 followed by LR model with 67.56% correctness and kappa value of 0.559 (Table. S3, Figs. S3–S6). While comparing both the model output with 2018 classified image reveals a significant, increase for woodlands by 4% (~14 Km^2^) (Fig. S2). The LR model predicts increment in the grassland cover by 1.8% (5.94 Km^2^), while using ANN predicted increase of 5.66% (18.31 Km^2^). Among the negative delta (-Δ) changes, a substantial shift has been predicted for shrubland cover of about −4.53% by ANN and −2.57 by LR, along with other transition types (Figs. S2, S4, S7 Tables S4–S6). Apart from the dominant land cover types, a significant decrease has been predicted by both future models in water coverage. Furthermore, considering the higher kappa value and correctness, the ANN output was selected for habitat suitability analysis for the future simulated landscape.

### Model performance and temporal change dynamics of suitability for rhino

A total of n = 42 spatially independent locations of rhino presence were selected by eliminating spatially correlated location by using SDM Toolbox Ver. 2. in ArcGIS 10.6. Out of 22 original sets of eco-geographic variables, only ten uncorrelated predictor variables were used for modelling (Table. S7). Model performance was good for all modelling approaches on both training and ten-fold cross-validation data (Table 1, Figs. S8–S10). The AUC (Area under the receiver operator curve) value of the models ranged between 0.95–0.81, the PCC, TSS, Cohen’s kappa, specificity and sensitivity also depicts decent performance of the models (Table 1, Fig. S10). Among all the models, the GLM and BRT retain five variables, MARS kept four, whereas the MAXENT and RF employed all the variables (Table 2). All five models reserved grassland cover as the most significant variable with contribution ranging between 0.39 and 0.18 (μAUC = 0.28), indicating its strong positive association with the habitat quality for *R*. *unicornis*. This was followed by the contribution of bare land and woodland ranging with μAUC = 0.16 and μAUC = 0.04, respectively (Table 2).

**Table 1:** Model fit metrics for each of the four species distribution modelling methods. Boosted Regression Tree (BRT), Random Forest (RF), Generalized Linear Model (GLM), and Generalized Additive Model (GAM). Model fit metrics included area under the receiver operator curve (AUC), Proportion Correctly Classified (PCC), True Skill Statistic (TSS), Cohen’s kappa, sensitivity, and specificity. Model fit was assessed on the training data used to fit the model as well as the withheld test data used for model evaluation.

METHOD	DATASET	AUC	Δ AUC	PCC	TSS	KAPPA	SPECIFICITY	SENCITIVITY
BRT	TRAIN	0.956	0.07	89.811	0.795	0.782	0.898	0.897
	CV	0.885 ± 0.11		83.454 ± 12.01	0.654 ± 0.234	0.652 ± 0.23	0.854 ± 0.17	0.8 ± 0.19
GLM	TRAIN	0.947	0.13	87.962	0.755	0.743	0.884	0.871
	CV	0.812 ± 0.17		76 ± 12.11	0.508 ± 0.311	0.478 ± 0.30	0.783 ± 0.15	0.725 ± 0.342
MARS	TRAIN	0.926	0.08	86.111	0.726	0.707	0.855	0.871
	CV	0.846 ± 0.12		77.818 ± 8.86	0.555 ± 0.19	0.535 ± 0.17	0.780 ± 0.15	0.775 ± 0.21
MAXENT	TRAIN	0.937	0.04	85.981	0.724	0.705	0.852	0.871
	CV	0.893 ± 0.08		84.181 ± 9.57	0.679 ± 0.20	0.665 ± 0.19	0.854 ± 0.15	0.825 ± 0.20
RF	TRAIN	0.881	0	82.407	0.646	0.629	0.826	0.82
	CV	0.881 ± 0.09		81.545 ± 5.93	0.636 ± 0.13	0.613 ± 0.11	0.811 ± 0.11	0.825 ± 0.16

**Table 2:** Variable importance using the increase in Area under the Curve (AUC) when each predictor variable is permuted using five different modelling environment to model habitat suitability of Rhino in GNP buffer forest. Area_am = Patch Area Distribution (area-weighted mean), Euclidian distance function from bare land landcover type = Bare land, Euclidian distance function from grassland landcover type = Grassland, Interspersion & Juxtaposition Index = Iji, Landscape Shape Index = Lsi, Number of Patches = NP, Euclidian distance function from river bank landcover type = Riverbank, Euclidian distance function from shrubland landcover type = Shrubland, Euclidian distance function from water = Water, Euclidian distance function from woodland landcover type = Woodland.

Variable Code	RF	BRT	GLM	MARS	MAXENT	µ AUC (Mean)
Area_am	0	—	—	—	0.003	0.001
Bare land	0.169	0.140	0.202	0.212	0.101	0.165
Grassland	0.189	0.242	0.393	0.301	0.283	0.281
Iji	0.019	—	—	0.019	0.034	0.024
Lsi	0	—	0.061	—	0	0.020
NP	0	—	—	—	0.004	0.002
River bank	0.006	0.006	—	—	0	0.004
Shrubland	0.001	0.015	0.055	—	0.002	0.024
Water	0.002	—	—	—	0	0.001
Woodland	0.001	0.022	0.123	0.069	0.032	0.049

#### Model performance and ensemble building

**Figure 3:**
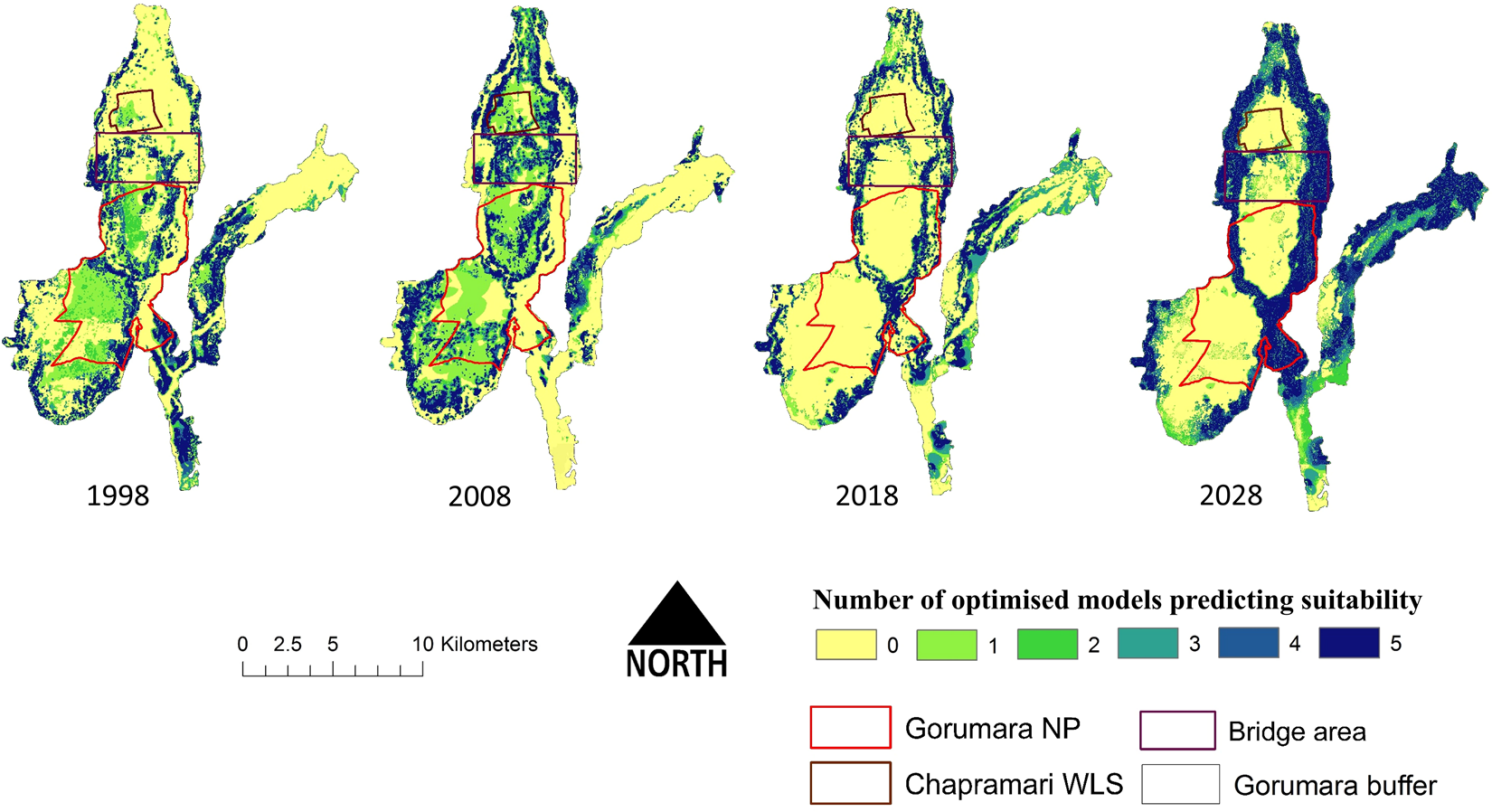
Maps indicating the number of participating models predicting habitat suitability for Rhino in GNP landscape of different decadal scenario, i.e. from the year 1998 (extreme left) to the year 2028 (absolute right). Each model surface displays a different threshold used to dichotomize continuous probabilities into a binary outcome. (Maps are generated using ArcGIS 10.6: www.esri.com).

For suitability model performance and ensemble mapping, the AUC score ranged from 0.95 to 0.88 for train and 0.88 to 0.81 for cross-validation split. The ΔAUC score ranged between 0 and 0.13, which indicated the models were sensitive to the data which was used for model fitting (Table 1). In terms of ΔAUC for cross-validation split, RF model had the best result with a score of 0, followed by MAXENT and BRT, giving ΔAUC values of 0.04 and 0.07 respectively (Table 1). Hence, by considering the AUC threshold (AUC > 0.7) of all the participating models, a combination of all models probabilities was used for developing the ensemble probability surface (Fig. S11) and ensemble count maps (Fig. 3).

#### Change in mean suitability for rhino

**Figure 4:**
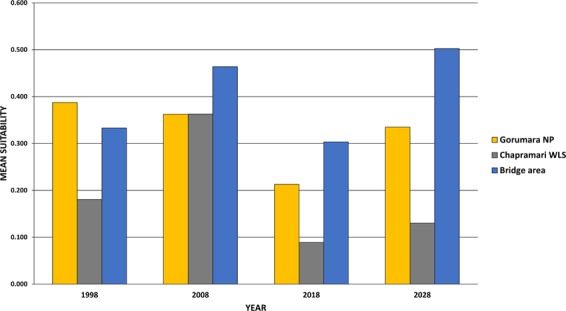
Decadal change in Mean Suitability area for rhino from the year 1998 to 2028. Mean suitability was computed in three zones, i.e. in two protected areas (GNP and CWLS) along with the bridge area outside the PAs.

Comparison of the mean suitability from the ensemble models generated by all temporal data sets reveals that the value in 1998 was highest for GNP, which was followed by territorial forest (bridge area) and least by CWLS. In 2008 mean values changed and became highest for bridge area (0.464), followed by both GNP and CWLS sharing a score of 0.363 (Fig. 4). In 2018 GNP and CWLS further showed a considerable decrease; however, bridge area have retained the highest value for mean suitability. In our predicted landcover for the year 2028, the model indicates that the suitable area for rhinos will increase up to 97.15 km^2^ which accounts for more than 13% increase from 2018 (Table. S8). However considering the fact that mean suitability of the bridge area in 2028 will going to be increased up to 0.503, which will be much higher than GNP (0.335) and CWLS (0.130), indicates that most of the preferred habitat resides in this particular non-protected zone.

#### Habitat quality estimation for rhino

Results of the mean patch area have shown a growing trend, as it has increased from 1998 to 2018 and a further increase has been predicted for 2028. In case of largest patch index (LPI) the major deviation was observed between the current (2018) and future 2028 showing increase of 16.79% in LPI of the *R*. *unicornis* suitable patches in the study landscape (Table 3). The decadal comparison in the Aggregation Index (AI) of the suitable habitat patches of *R*. *unicornis* indicating increasing trends (Table 3).

**Table 3:** Quality estimation of suitable area for rhino based on ensemble models. Change in fragmentation of rhino suitable habitat in the GNP landscape during four scenarios, i.e., in 1998, 2008, 2018 and 2028. Table show metrics quantifying the area and fragmentation of grid cells in the ensemble model where the rhino was projected to be present, including total area Class Area (CA), mean patch area (AREA_MN), Largest Patch Index (LPI) and Aggregation Index (AI).

Year	CA (ha)	PD (no. of patches/100 ha)	LPI %	AREA_MN (ha)	AI %
1998	5541.75	3.272	2.509	5.233	79.544
2008	6059.34	3.350	2.179	5.689	80.149
2018	5327.19	2.844	2.250	5.790	81.058
2028	9715.23	1.469	19.045	20.803	84.738

## Discussion

The Indian rhino (*Rhinoceros unicornis*), a mega-herbivore of Himalayan foothill and flood plains, is adapted to a mosaic of tall grasslands and riverine forests habitats where water and green growth remains available throughout the year^48,49^. The species has faced significant setback due to habitat loss and illegal poaching for its horn^1^. This resulted in a decline in much of its historical range and confinement to only a few Protected Areas (PAs) of Assam, West Bengal in India and Nepal, besides a small reintroduced population in Dudhwa National Park, Uttar Pradesh in India^7^.

The present multi-temporal habitat change analysis revealed change in configuration of the habitat and its impact on the suitability for rhino. The grassland cover area during the three decades has changed significantly in the region and found to decrease within the limits of the GNP. The decrease in grassland cover in the PA, resulted in movement of *R*. *unicornis* outside the PA in search of food leading to increasing in rhino-human conflicts in the landscape. The habitat ecology studies on rhinos found that the species prefer grasslands over other types of habitats with seasonal variability^9,13,50–52^ and supports our findings. Hence, it is imperative to manage the intactness of the grasslands for the longer viability of rhino and may also contribute to reducing conflicts in the surrounding areas of the GNP. Furthermore, the grassland patches in the forest range of the territorial division which surrounds GNP are vital for the long term viability of the rhino population.

Further, the comparative transition probability matrices brought out that during the last two decades (1998–2018) bare land was converted to shrubland and grassland to shrubland cover. The development of more scrublands in the bridge areas may also contribute towards conservation of other species in the park, i.e. leopard, which are known to excellently adapt to such shrubland interface areas because of plasticity^53^.

The simulation of the future land cover for the year 2028 by both ANN and LR algorithms, indicated an increase of woodland cover mainly in PA, while the ANN showed an increment of grassland in the study landscape mostly in the territorial forest ranges outside GNP. This indicates the increase of feeding grounds and suitable habitat for rhino outside the PA network in the landscape. Moreover, in PAs (GNP, CWLS) the transformation of shrubland and bare land cover to woodland over the two decades resulting in an increment of the woodland cover area, this transformation can be attributed to plantation activities by the local forest management agency.

Among all the participating model’s grassland has been retained as the most significant variable (μAUC = 0.28), indicating its strong association with the habitat quality for rhino^20^. Further, the study revealed that not only the habitat suitability will increase in simulated 2028 by 13% (Table. S8) moreover the mean patch area of the suitable habitats will also increase up to 15 ha in the year 2018. The predicted increase of suitable patches and their configuration indicated improvement in habitat quality^54^. Furthermore, anticipated growth in the aggregation index (AI) means an enhancement in continuation of suitable patches in the landscape.

## Conclusion

In the northern region of the West Bengal state habitat fragmentation and loss has resulted in the confinement of the rhino populations into two isolated PAs, namely JNP and GNP. The PA network, along with the territorial forest in the region holds viable populations of three mega-herbivores including rhino, elephant and gaur. The *R*. *unicornis* in GNP and associated forests are adapted to feed mainly on a wide variety of grasses and hence prefer grassland cover more than other types of habitats. Thus, the management of grasslands and riverine habitat management are crucial for maintaining the population viability in the landscape.

Due to actions of the forest department, the poaching pressures have decreased, which has resulted in a considerable increment in the rhino population, i.e. up to 50 individuals in GNP^55^. Moreover, improvement in the other populations of rhino in India and Nepal resulted in down gradation of its IUCN conservation status from ‘Endangered’ to ‘Vulnerable’ in 2008.

However, due to increase in populations of sympatric mega-herbivores, i.e. largely *Bos gaurus* (gaur) [900+ individuals in GNP] and *Elephas maximus* (elephant) in Gorumara landscape, followed by excessive cattle grazing pressure, habitat degradation and fragmentation resulting in exceeded carrying capacity of the landscape for *R*. *unicornis* and other mega-herbivores. One of the fundamental factors behind the increase in the population of these species maybe because of the absence of top predators, i.e. tigers^56,57^.

The GNP is a safe abode for *R*. *unicornis* but possesses only 10% grassland cover of the total area which seems not sufficient for holding *R*. *unicornis* hence they frequently move outside the park to meet their foraging needs^15^. Furthermore, these remaining grassland patches in GNP are acting as interspecific competition grounds for other mega-herbivores, particularly the gaur^21^.

The increment in the suitability of *R*. *unicornis* in the territorial range in the last two decades as well as predicted for 2028 is posing serious conservation and management challenges to the managers. Since, this territorial range or the bridge area is under anthropogenic pressures such as railways, road network and other developmental activities which may threaten the rhino population to a greater extent. Furthermore, the bridge area may expose *R*. *unicornis* and other species to poachers because *R*. *unicornis*, in particular, tend to move from the regions of low suitable patches to high suitable patches^20^.

Hence, through the present analysis, we wanted to highlight that there is an urgent need to manage the population of *R*. *unicornis* and other species by adopting core principals of landscape ecology. We also suggest that the two PAs, i.e. GNP and CWLS may be united by bringing the bridge area (territorial forest) into the PA area to facilitate long term conservation and management of mega-herbivores in the landscape. The joining of PAs may reduce habitat fragmentation and will be of significant value for minimising growing human-wildlife conflicts. The impacts of linear features such as highways and railway lines can be mitigated by adopting best practices on eco-friendly mitigation measures for connecting landscapes. The proposals include the development of overpasses, underpass, bridge, ecoduct and viaducts for providing safe passage to mega-herbivores and other species are found to be useful in conservation and management of wildlife species^58^.

## Materials and Methods

### Study area

The Gorumara landscape is situated in the northern part of the Indian state of West Bengal between latitude 26°47′12.5′′N to 26°43′25.6′′N and longitude 88°52′4.2′′E to 88°47′7.3′′E (Fig. 1). The total area of the study landscape is about 323.26 Km^2,^ and it belongs to the biogeographical zone 7B-Lower Gangetic Plain^59^. The study landscape is a mosaic of PAs, territorial forest, agriculture and human habitations. The PAs includes GNP and CWLS which fall under Gorumara Wildlife Division, and territorial forest range, which falls under Jalpaiguri Division. Due to its position in the foothills of Central Himalayas, the area is rich in biological diversity. The landscape is home to about 48 species of mammals (carnivores and herbivores), 193 species of birds, 22 species of reptiles, 40 species of fishes and other macro and microfauna^60^. The Gorumara landscape is known for a natural population of Indian Rhinoceros (*Rhinoceros unicornis*), along with other mega-herbivores such as Asian Elephant (*Elephas maximus*) and Gaur (*Bos gaurus*). The terrain of the landscape can be differentiated into a distinct plateau and plains with little undulations. The soil of the area is commonly alluvial with bhabar formations^60^. The water cover in the landscape is represented by a river system of three main rivers Murti, Indong and Garati which drains into Jaldakha River.

### Field data collection

The primary data was collected during the year 2016–2018. After the reconnaissance survey, nine permanent linear transects of varied length 3 km to 6 km were laid systematically in the entire landscape representing all different habitat variability of the GNP and CWLS. Along the study transect, a total of 118 nested sampling plots of the different radius (10 m radius plot for the tree, 5 m radius plot for shrubs and 1 × 1 m quadrates for ground vegetation) were laid at regular intervals for recording vegetation type and plant species composition^61,62^. The *R*. *unicornis* direct, as well as indirect evidence such as digging signs, presence of dung and hoof marks, browsing sign, were recorded on the transects systematically. All transects were sampled twice in all three seasons (pre-monsoon, post-monsoon and winter) during 2016–18. The field survey resulted in the documentation of n = 57 presence records for *R*. *unicornis*. For all presence location of rhino information such as GPS location, habitat type, terrain type, distance to water, distance to the road, and disturbance was recorded.

### Landsat data collection and pre-processing

For understanding the change in land cover between 1998 and 2018 in the study area, the Landsat images data of the year 1998, 2008 and 2018 were downloaded from USGS (https://earthexplorer.usgs.gov). Due to the difference between the TM (Thematic Mapper) and OLI (Operational Land Imager) sensor, the geometric correction for the year 1998 was done using the DEM (Digital elevation model) data followed by georeferencing with 2008 and 2018 image for terrain correction^63,64^ and all images were scaled for a spatial resolution of 30 m. The geometrically rectified images were then processed for Top of Atmosphere (TOA) Reflectance (combined surface and atmospheric reflectance) in order to reduce the in-between-scene variability through a normalisation process for solar irradiance with a combination of DOS1 (Dark Object Subtraction 1) corrections^65^.

### Land cover classification and accuracy estimation

The land cover classification of the three different decadal images (i.e. for year 1998, 2008 and 2018) was carried out by adopting the Maximum likelihood technique of supervised classification by using Semi-Automatic Classification Plugin of QGIS^66^. A total of six dominant land cover classes were identified namely Water; Riverbank; Bare land; Woodland, Grassland and Shrubland. The training data for 2018 Landsat image was collected for all the six land cover classes during the field surveys in the study landscape. The training data was composed of 165 polygons comprising 1566 pixels for the 2018 image. Further, for the rest of the two decadal year images of 1998 and 2008, the classified image of 2018 was used as reference material^63^. We have used a similar classification approach for the classification of 1998 and 2008 decadal images by overlaying the training data for 2018 on 1998 and 2008 images. This was followed by the selection and elimination process for training samples which showed a change in cover^63,64^. The classified image of the year 2018 was selected to check the accuracy based on the ground-truthing data collected during the field visit. The overall accuracy, user and producer accuracy along with the kappa coefficient were then derived from the error matrices^67,68^. All image related classification, as well as accuracy assessments, were carried out using the semi-automatic Classification plugin of QGIS, ENVI 5.1 and ESRI ArcGIS 10. 6.

### Land cover simulation for the year 2028 and change dynamics analysis

We have used Artificial Neural Network (ANN) and Logistic Regression (LR) methods to model land cover transition potential. The LR is being used to predict the probability of occurrence of a particular event by the values of a set of features whereas the ANN uses backpropagation gradient calculation method which updates the weights of a multilayer perceptron^69,70^. The MOLUSCE plugin of QGIS was used to predict future land cover following the cellular automata (CA) model. As MOLUSCE only work with raster data, all vector data sets were converted into raster, resampled at 30 × 30 m cell and were projected at WGS_1984_UTM_ZONE_ (45 N). For projecting the simulated results, the cellular-automata simulation was used, based on the Monte Carlo algorithm^69,71–74^. The simulated map for the year 2028 was based on classified images of 2008 and 2018. Our prediction for the future accounts only the change that has happened previously without taking catastrophic events into consideration. A total of seven variables were used as land-use drivers (Table 4). The drivers/predictor variables for future land cover simulation were grouped into three categories, namely, Surface texture/configuration drivers, climatic drivers and anthropogenic drivers (Table 4). The topographic variables were used as surface texture/configuration drivers. Heat load index for temperature^75^ and integrated Moisture Index^76^ drivers were generated as a proxy for bioclimatic drivers. Linear features such as rail and road networks were considered as the anthropogenic variables. The data on these variables were prepared using the Geomorphometric and Gradient Metrics Toolbox Ver. 2.0^77^ of ArcGIS 10.6. For validation of simulated land cover of 2028, observer and producer accuracy along with kappa statistics were computed. The total area change in Km^2^ along with transition potential of respective landcover categories for future landscape images, was analysed.

**Table 4:** Variables used for landscape modelling. The topographic variables were used as surface texture/configuration drivers, temperature and moisture drivers as a proxy for bioclimatic drivers, and the influence of rail and road development was considered as the anthropogenic variables. Geomorphometric and gradient metrics toolbox were used for the calculation of matrices and processed in ArcGIS 10.6.

Variables	Abbreviation	TYPE
Compound topographic index	CTI	Continuous
Integrated Moisture Index	IMI	Continuous
Heat load index	HLI	Continuous
Linear aspect	LA	Continuous
Euclidian distance function from road	EU_ROAD	Continuous
Euclidian distance function from rail	EU_RAIL	Continuous
Roughness	RH	Continuous

### Model preparation, evaluation and change dynamics of ensemble models

The habitat suitable for rhino was mapped using the five different modelling algorithms namely; Generalized Linear Model (GLM), Multivariate Adaptive Regression Splines (MARS), Boosted Regression Trees (BRT), Random Forest (RF) algorithm, and Maximum Entropy (Maxent), for the years 1998, 2008, 2018 and 2028 (Fig. S12)^78–80^.

The selection of predictors plays a significant role in determining the habitat of species; hence, we selected those variables which are ecologically important for the study species^81–83^. Out of the total 22 predictors selected as potential candidates for identifying the suitable habitats of rhino in GNP (Table S9). Out of which only 10 variables (Table S7) were selected, after multi-collinearity testing. We have used Pearson Correlation Coefficients (r) with a threshold of (r) < 0.8 for variable selection, using the SAHM package^84^.

The eco-geographical variables were used and categorised at two scales, i.e. Land cover class-level variables and Landscape-level variables. The land cover class-level variables were generated by vectorisation of the land cover classes followed by creating euclidian distance using ArcGIS 10.6. Whereas the landscape-level metrics were computed by using the moving window function of FRAGSTATS Ver. 4.2 software. Square shape window with a side length of 100 m was used, and 8-cell neighbour rule was applied for all standard analysis. The FRAGSTATS was used by several studies for understanding the configuration of the landscape^85,86^. After making the model for the year 2018, we projected the same for past decades year 1998, 2008 and also for the future year 2028. All the variables were re-sampled at 30 m resolution and were converted to ascii (raster) format using the spatial analyst extension of ArcGIS 10.6^87^. The threshold value based on the AUC of the ROC ranges from 0 to 1, the AUC score of 1 indicates perfect prediction, with zero omission.

The ensemble count maps (ranged from 0–5) further were made binary by considering the number of the optimised model predicting the habitat suitability for rhino. The highest value of model agreement five (5) was considered for the comparison in our multi-temporal ensemble approach. A number of studies are available, highlighting the robustness of the ensemble modelling approach in predicting the suitable habitats precisely^78–80,88–91^. All the individual models resulted in a probability surfaces indicating the suitable habitat for rhino in the study landscape. The probability surfaces were generated using minimum training presence as a threshold for the respective models^92^.

For comparing the performances and selection of models, AUC (area under the receiver operator curve) was used due to its wide acceptance in SDM studies^93,94^. The ensemble was created using the models with AUC > 0.7^94^, and our ensemble models were the running averages of the binary estimates of the participating models with a value of 0 or 1. For model fitting and also to calculate the related performance metrics, SAHM package for VisTrails software was used^95,96^. We examined the appropriateness of model complexity by examining the difference between AUC-train and AUC-CV (ΔAUC) to provide information on how sensitive the model is to the data which is being used to fit it^94^. Moreover, True Skill Statistic (TSS)^97^, Cohen’s Kappa^98^, Proportion Correctly Classified (PCC), specificity, and sensitivity was also estimated for training and cross-validation (n = 10)^99–104^. Mean AUC (µ AUC) value for each predictor variables was calculated for each model, followed by ranking predictors variables based on µ AUC (Mean)^94,99^. Variable importance was calculated using an increase in AUC after fitting the final model with and without each predictor variables in the final model^103^. Predictors for rhino’s habitat suitability were then ranked based on the µ AUC (Mean).

For both the present and future scenarios, the FRAGSTAT Ver. 4.2^104^ along with zonal statistics calculation in ArcGIS 10.6 were used to estimate the area of habitat suitable for the species and also the degree of fragmentation in the suitable habitat. For the present study Total area (TA), Mean patch area (MPA), Largest Patch Index (LPI) and Aggregation Index (AI), were computed to understand the level of fragmentation in suitable habitat across multi-temporal scenarios. Finally, the zonal statistics were calculated for estimating the change in *R*. *unicornis* suitable habitat within the PAs, i.e. GNP and CWLS and also in the territorial forest range including the bridge area.

## Supplementary information

Supporting Information.
