# Supplementary material for: Past, Present and Future: Combining habitat suitability and future landcover simulation for long-term conservation management of Indian rhino
Source: Sci Rep. 2020 Jan 17;10:606. doi: 10.1038/s41598-020-57547-0 (PMC6969075; doi:10.1038/s41598-020-57547-0)
Supplement: Supplementary file 1 — Supporting Information. [file 41598_2020_57547_MOESM1_ESM.pdf]

Supplementary information for article titled:

Past, Present and Future: Combining habitat suitability and future landcover simulation for long-term conservation management of Indian rhino

Authors: Tanoy Mukherjee, Lalit Kumar Sharma, Goutam K. Saha, Mukesh Thakur, Kailash Chandra

Supplementary Table. S1 Accuracy assessment table for land cover classification of study area for the year 2018.

**Land cover classification for GNP**									
**Contingency Matrix**									
		**Observed land cover Classes**						**Grand Total:**	**User's Accuracy:**
		**Water**	**Bare land**	**River bank**	**Grassland**	**Shrubland**	**Woodland**		
**Mapped land cover Classes:**	**Water**	56	0	6	0	0	0	61	91.803
	**Bare land**	1	52	3	3	0	0	64	81.250
	**River bank**	3	5	51	1	0	0	55	92.727
	**Grassland**	0	0	0	49	4	2	54	90.741
	**Shrubland**	0	3	0	7	53	2	68	77.941
	**Woodland**	0	0	0	0	3	56	58	96.552
**Grand Total:**		60	60	60	60	60	60	60	
**Producer's Accuracy:**		93.333	86.667	85.000	81.667	88.333	93.333		
**Samples: 360 Overall Accuracy: 88.05% Kappa Statistic: 85.70%**									

Kappa Coefficient
Number of observed agreements: 317 (88.06% of the observations)

Number of agreements expected by chance: 60.0 (16.67% of the observations)

Kappa= 0.857

SE of kappa = 0.021

95% confidence interval: From 0.816 to 0.897

The strength of agreement is considered to be 'very good'.

The calculations above only consider exact matches between observers. If the categories (A, B, C...) are ordered, you may also wish to consider close matches. In other words, if one observer classifies a subject into group B and the other into group C, this is closer than if one classifies into A and the other into D. The calculation of weighted kappa, below, assumes the categories are ordered and accounts for how far apart the two raters are. This calculation uses linear weights.

Weighted Kappa= 0.910

Supplementary Figure S1. Land cover of all six different classes in Km^2 area i.e. for Water, River bank, Bareland, Woodland, Grassland and Shrubland in years 1998, 2008, 2018 and 2028. Different colour code have been used to visualize different decadal images.

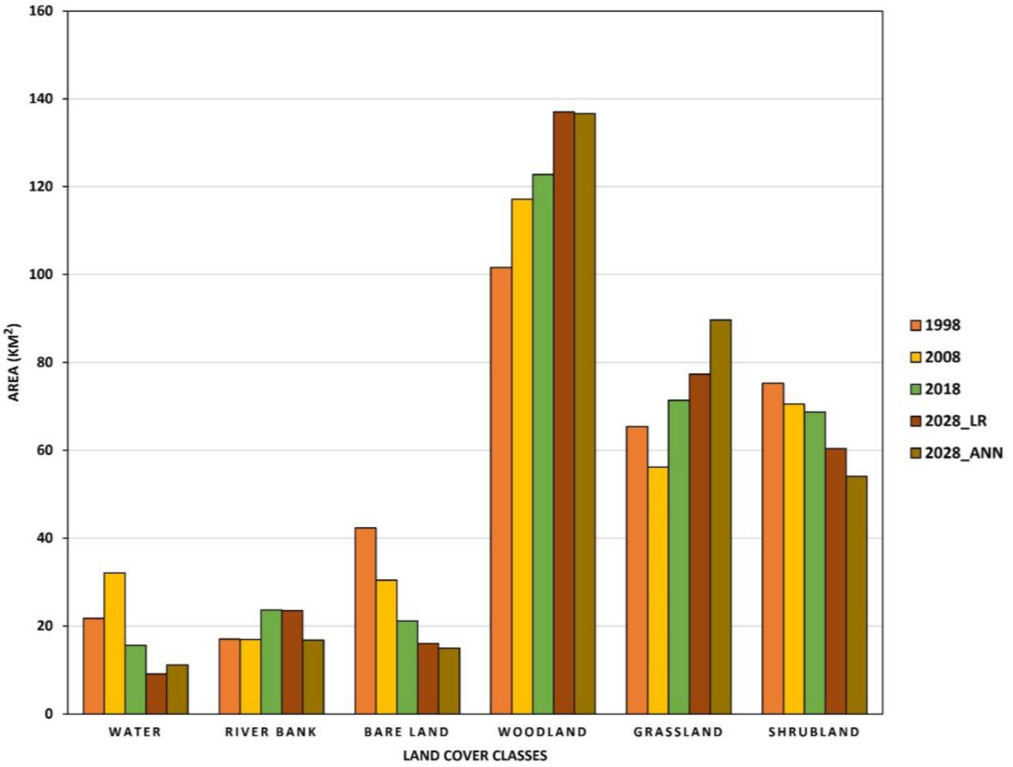

Supplementary Figure S2. Delta change in percentage of land cover of all six different classes i.e. in Water, River bank, Bareland, Woodland, Grassland and Shrubland in years 1998, 2008, 2018 and 2028. Different colour code have been used to visualize different landcover types.

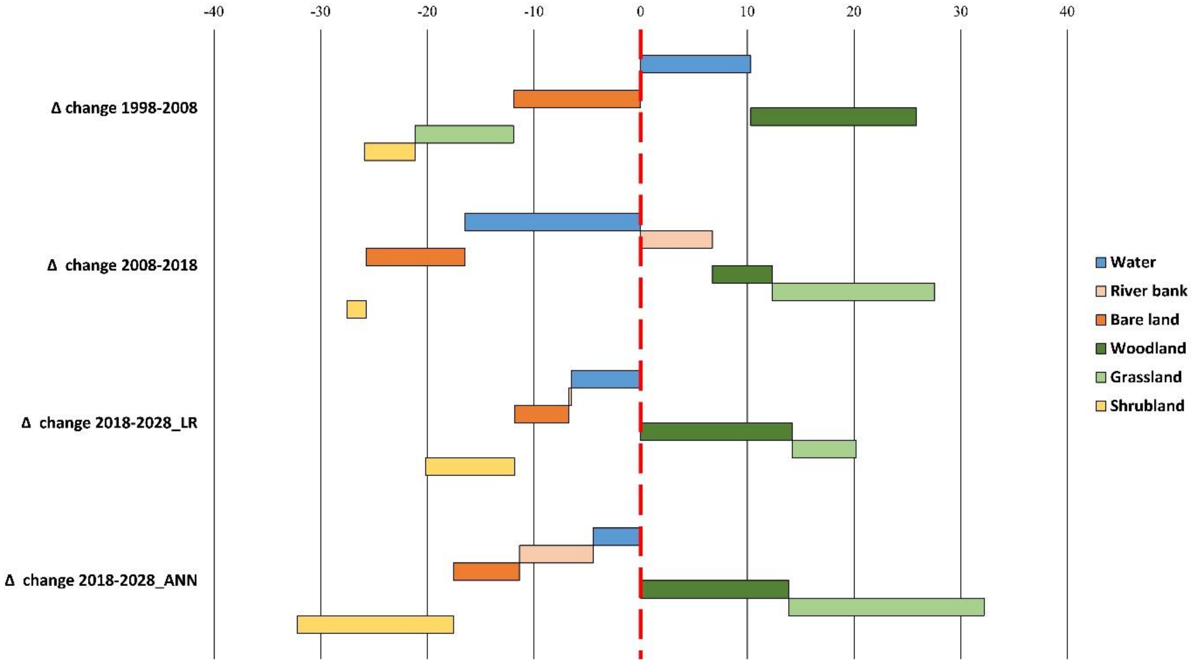

Supplementary Table S2. Transitional probability matrix of Landcover change from the year 1998-2008 and from 2008-2018.

Transition period	Land Cover types						
**1998-2008**	**To**	Water	River bank	Bare land	Woodland	Grassland	Shrubland
**From**	Water	0.4521	0.2564	0.0425	0.0060	0.0069	0.2361
	River bank	0.4780	0.3315	0.1054	0.0000	0.0019	0.0833
	Bare land	0.1604	0.0823	0.2204	0.0026	0.0256	0.5087
	Woodland	0.0022	0.0006	0.0045	0.8681	0.1182	0.0064
	Grassland	0.0583	0.0271	0.0144	0.2328	0.3259	0.3415
	Shrubland	0.0436	0.0057	0.2261	0.1792	0.2871	0.2584
**2008-2018**	**To**						
**From**	Water	0.2404	0.3657	0.1034	0.0014	0.2531	0.0359
	River bank	0.1458	0.5381	0.1956	0.0000	0.1055	0.0150
	Bare land	0.0483	0.0131	0.1778	0.0237	0.1674	0.5696
	Woodland	0.0020	0.0002	0.0001	0.8796	0.0269	0.0912
	Grassland	0.0084	0.0093	0.0029	0.3155	0.2971	0.3669
	Shrubland	0.0462	0.0268	0.1271	0.0173	0.5178	0.2648

Supplementary Table S3. Validation of both ANN an LR model for 2028 landscape prediction**.** Showing the correctness percentage along with Kappa statistics for both ANN and LR stimulated models.

**Validation matrix**	**ANN**	**LR**
**% of correctness**	82.10949	67.56809
**Kappa (overal)**	0.75635	0.55978
**Kappa (histo)**	0.8644	0.9153
**Kappa (loc)**	0.875	0.61159

Supplementary Figure. S3. Learning curve for Artificial Neural Network (ANN). Where training data is represented by green and validation data has been represented by red colour.

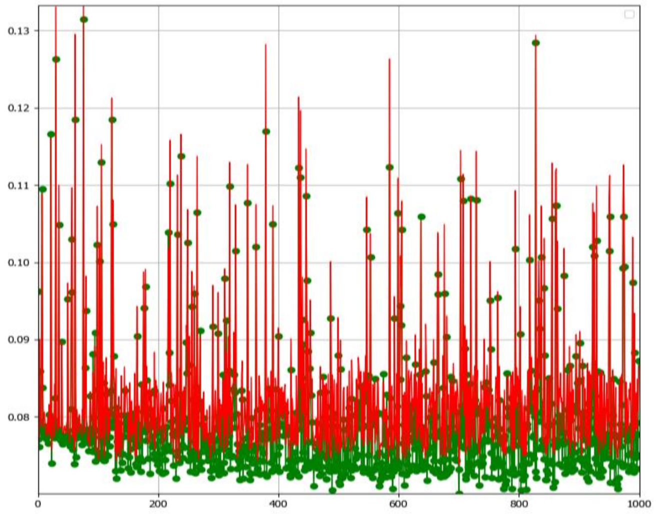

Supplementary Figure. S4. Validation through multiple resolution budget for ANN stimulated landcover of 2028. After stimulation of future landscape for 2028 by ANN model quality have been assed based on various assessment on the similarity between the forecast and the true state of the territory. Green- represents no location, no quantity inform; Purple- represents medium location, medium quantity inform; Yellow- represents perfect location, perfect quantity inform; Red-represents no location, medium quantity inform; Black- represents perfect location, medium quantity inform.

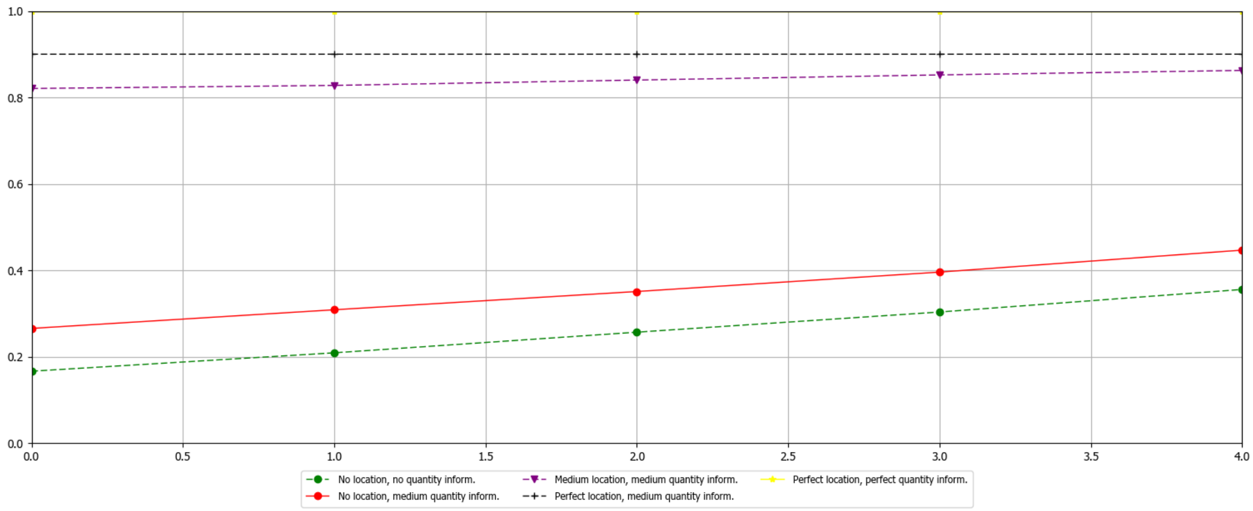

Supplementary Figure. S5 Validation through multiple resolution budget for LR stimulated landcover of 2028. After stimulation of future landscape for 2028 by LR model quality have been assed based on various assessment on the similarity between the forecast and the true state of the territory. Green- represents no location, no quantity inform; Purple- represents medium location, medium quantity inform; Yellow- represents perfect location, perfect quantity inform; Red-represents no location, medium quantity inform; Black- represents perfect location, medium quantity inform.

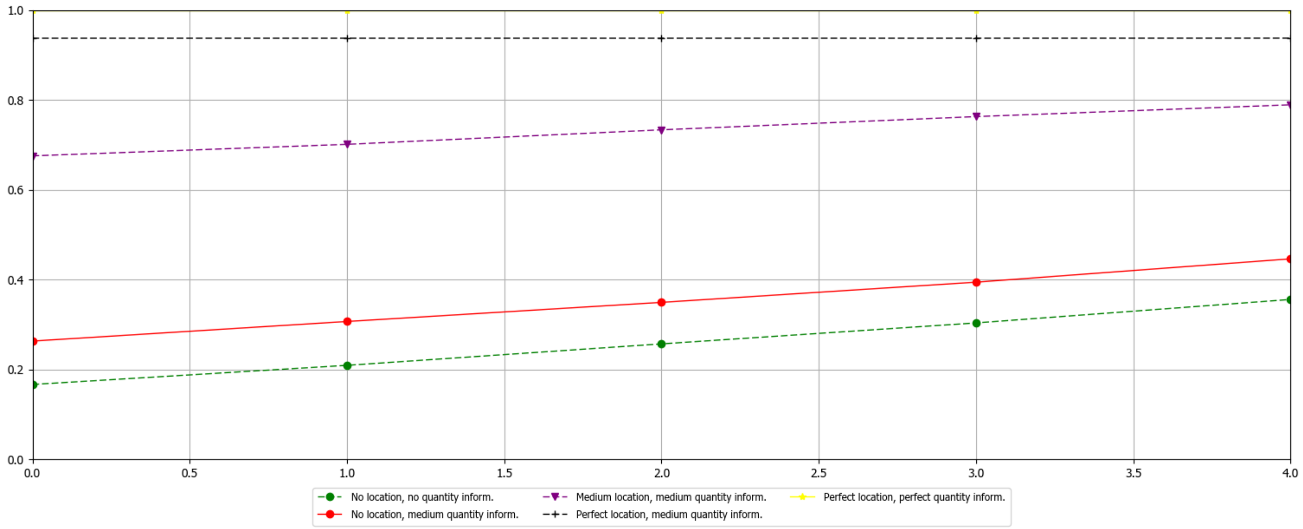

Supplementary Figure. S6 Certancy function representation of ANN in left and LR in right. Represents a confidence map in the forecast, measured in percentage ranges from 0-100; where 100= complete confidence and no other alternative forecast are insignificant and complete 0 means = complete uncertainty showing, with at least one or more alternative with the same likelihood of realization. a. Represents the certancy function for LR model b. represents the certancy function of ANN model. All maps were generated using ArcGIS Ver. 10.6.

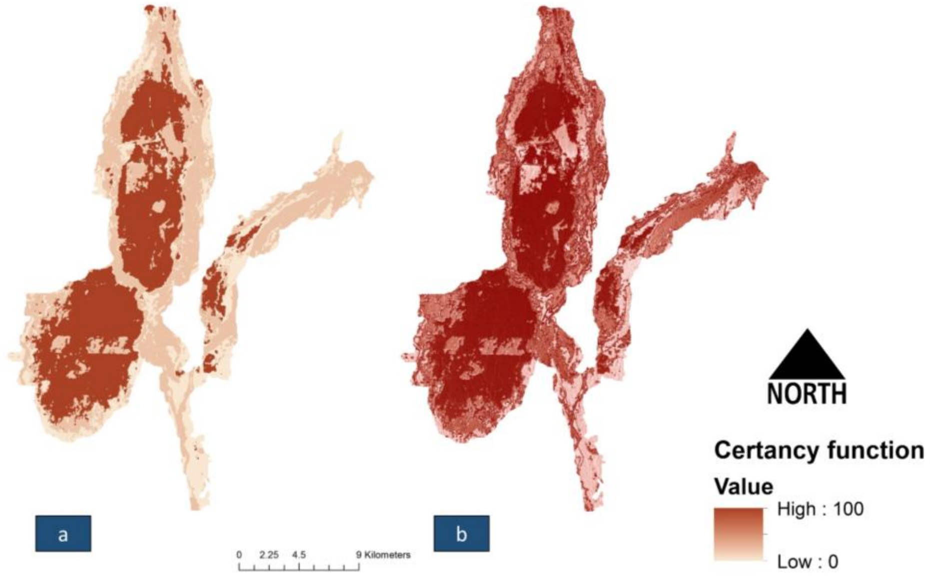

Supplementary Table S4. Transitional probability matrix of Landcover change from the year 2018-2028 stimulated by logistic regression (LR) model and from 2018-2028 Artificial neural network (ANN) stimulated model.

**Transition period**							
**2018-2028 (LR)**	To	Water	River bank	Bare land	Woodland	Grassland	Shrubland
**From**	Water	0.290492	0.324511	0.037998	0.001614	0.269042	0.076342
	River bank	0.106275	0.721247	0.093778	0.000646	0.06909	0.008964
	Bare land	0.044431	0.017671	0.373545	0.024509	0.080877	0.458967
	Woodland	0.000183	0.000645	0.000403	0.957844	0.013422	0.027503
	Grassland	0.001916	0.000252	0.000593	0.248112	0.552985	0.196142
	Shrubland	0.013985	0.012243	0.076273	0.016682	0.416839	0.463978
**2018-2028 (ANN)**							
	Water	0.715332	0	0	0.004152	0.276711	0.003806
	River bank	0	0.707802	0	0.000304	0.291477	0.000418
	Bare land	0	0	0.708776	0.000637	0.289653	0.000935
	Woodland	0	0	0	0.96922	0.00041	0.030369
	Grassland	0	0	0	0.235706	0.755645	0.008649
	Shrubland	0	0	0	0.011038	0.266793	0.722168

Supplementary Table S5. Representing all transition types in hectors (ha) from one cover type to another from 2018 to ANN stimulated 2028 landcover.

	Transition type	Area Hectare (ha)
1.	Water to Water	1116.54
2.	Water to Woodland	6.48
3.	Water to Grassland	431.91
4.	Water to Shrubland	5.94
5.	Riverbank to Riverbank	1677.15
6.	Riverbank to Woodland	0.72
7.	Riverbank to Grassland	690.66
8.	Riverbank to Shrubland	0.99
9.	Bareland to Bareland	1501.74
10.	Bareland to Woodland	1.35
11.	Bareland to Grassland	613.71
12.	Bareland to Shrubland	1.98
13.	Woodland to Woodland	11899.98
14.	Woodland to Grassland	5.04
15.	Woodland to Shrubland	372.87
16.	Grassland to Woodland	1682.55
17.	Grassland to Grassland	5394.06
18.	Grassland to Shrubland	61.74
19.	Shrubland to Woodland	75.87
20.	Shrubland to Grassland	1833.75
21.	Shrubland to Shrubland	4963.68

Supplementary Table S6 Representing all transition types in hectors (ha) from one cover type to another from 2018 to (LR) stimulated 2028 landcover.

	Transition type	Area Hectare (ha)
1.	Water to Water	453.42
2.	Water to Riverbank	506.52
3.	Water to Bareland	59.31
4.	Water to Woodland	2.52
5.	Water to Grassland	419.94
6.	Water to Shrubland	119.16
7.	Riverbank to Water	251.82
8.	Riverbank to Riverbank	1709.01
9.	Riverbank to Bareland	222.21
10.	Riverbank to Woodland	1.53
11.	Riverbank to Grassland	163.71
12.	Riverbank to Shrubland	21.24
13.	Bareland to Water	94.14
14.	Bareland to Riverbank	37.44
15.	Bareland to Bareland	791.46

**16.**	Bareland to Woodland	51.93
**17.**	Bareland to Grassland	171.36
**18.**	Bareland to Shrubland	972.45
**19.**	Woodland to Water	2.25
**20.**	Woodland to Riverbank	7.92
**21.**	Woodland to Bareland	4.95
**22.**	Woodland to Woodland	11760.3
**23.**	Woodland to Grassland	164.79
**24.**	Woodland to Shrubland	337.68
**25.**	Grassland to Water	13.68
**26.**	Grassland to Riverbank	1.8
**27.**	Grassland to Bareland	4.23
**28.**	Grassland to Woodland	1771.11
**29.**	Grassland to Grassland	3947.4
**30.**	Grassland to Shrubland	1400.13
**31.**	Shrubland to Water	96.12
**32.**	Shrubland to Riverbank	84.15
**33.**	Shrubland to Bareland	524.25
**34.**	Shrubland to Woodland	114.66
**35.**	Shrubland to Grassland	2865.06
**36.**	Shrubland to Shrubland	3189.06

Supplementary Figure. S7 Transition potential showing different transition types in study area. With different colour ramp indicates different transition types in the study landscape from the year 2018 to 2028. a. Represents transition from one cover type to another from 2018 to ANN model for 2028. b. Represents transition from one cover type to another from 2018 to LR model for 2028. All maps were generated using ArcGIS Ver. 10.6.

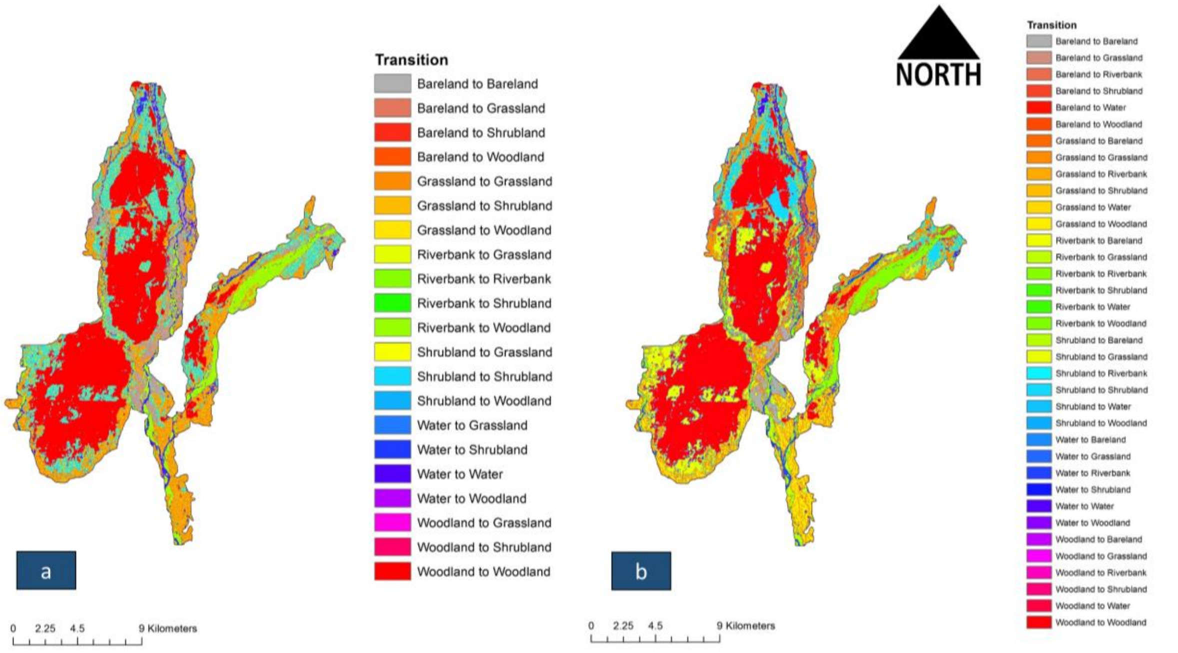

Supplementary Table S7. List of selected eco geographic variables used for suitability modeling. Eco-geographical variables were categorised into two scales i.e. Land cover class-level variables and Landscape-level variables. The landcover class-level variables were generated by vectorization of the landcover classes followed by generating euclidian distance using ArcGIS 10.6 software. Whereas the landscape-level metrics were computed by using the moving window function of FRAGSTAT Ver. 4.2 software.

**Variables**	**Code**	**Type**
**Landscape-level variables**		
Area (area-weighted mean)	area_am	Continuous
Euclidian distance function from bare land landcover type	Bare_land	Continuous
Euclidian distance function from grassland landcover type	Grassland	Continuous
Interspersion and Juxtaposition Index	Iji	Continuous
Landscape Shape Index	lsi	Continuous
Number of patches	np	Continuous
**Land cover class-level variables**		
Euclidian distance function from river bank landcover type	River_bank	Continuous
Euclidian distance function from shrubland landcover type	Shrubland	Continuous
Euclidian distance function from water	Water	Continuous
Euclidian distance function from woodland landcover type	Woodland	Continuous

Supplementary Figure S8. Model evaluation plot, showing the average training ROC of both training and cross-validation (CV) for the replicate runs under 5 models where **a.** showing ROC plot of boosted regression tree (BRT) **b.** generalized linear model (GLM), **c.** multivariate adaptive regression spines (MARS), **d.** Maximum entropy (MaxEnt), **e.** Random forest (RF).

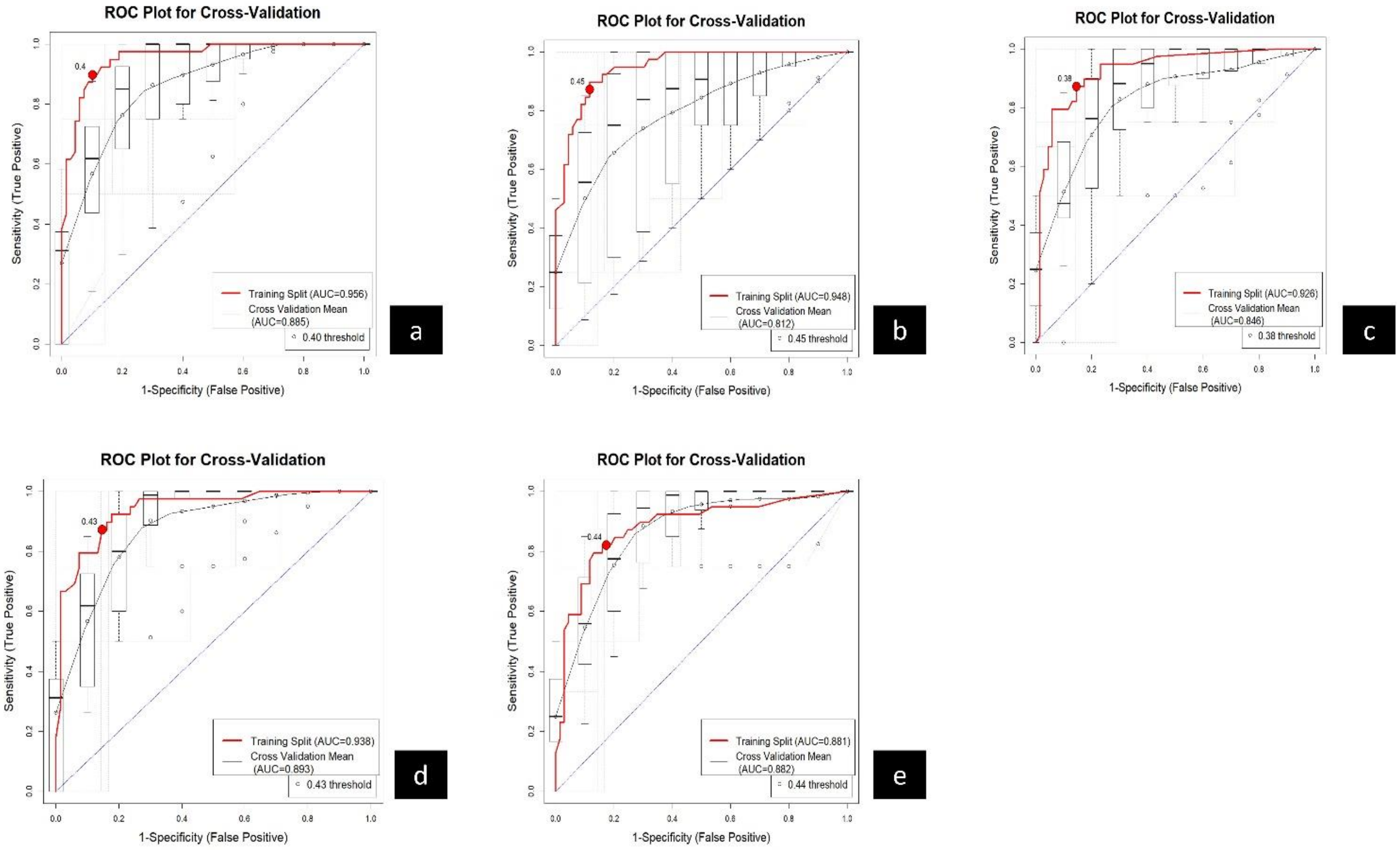

Supplementary Figure. S9. Confusion matrixes, Model Calibration plots and Residual plots. **Row 1** represents spatial pattern of residuals where colour ramp indicates the magnitude of deviance and size represents the quantity. **Row 2** represents model calibration plot across all 5 different model for cross-validation split. **Row 3** represents confusion matrix for all 5 models, plotted by observed vs. predicted where colour ramp from lowest value 0% (white) to 100% (red) indicates the quantification of particular pair types. **Column a**. represents plots for BRT, **Column b**. represents plots for GLM, **Column c**. represents plots for MARS, **Column d**. represents plots for MaxEnt and **Column e**. represents plots for e. RF.

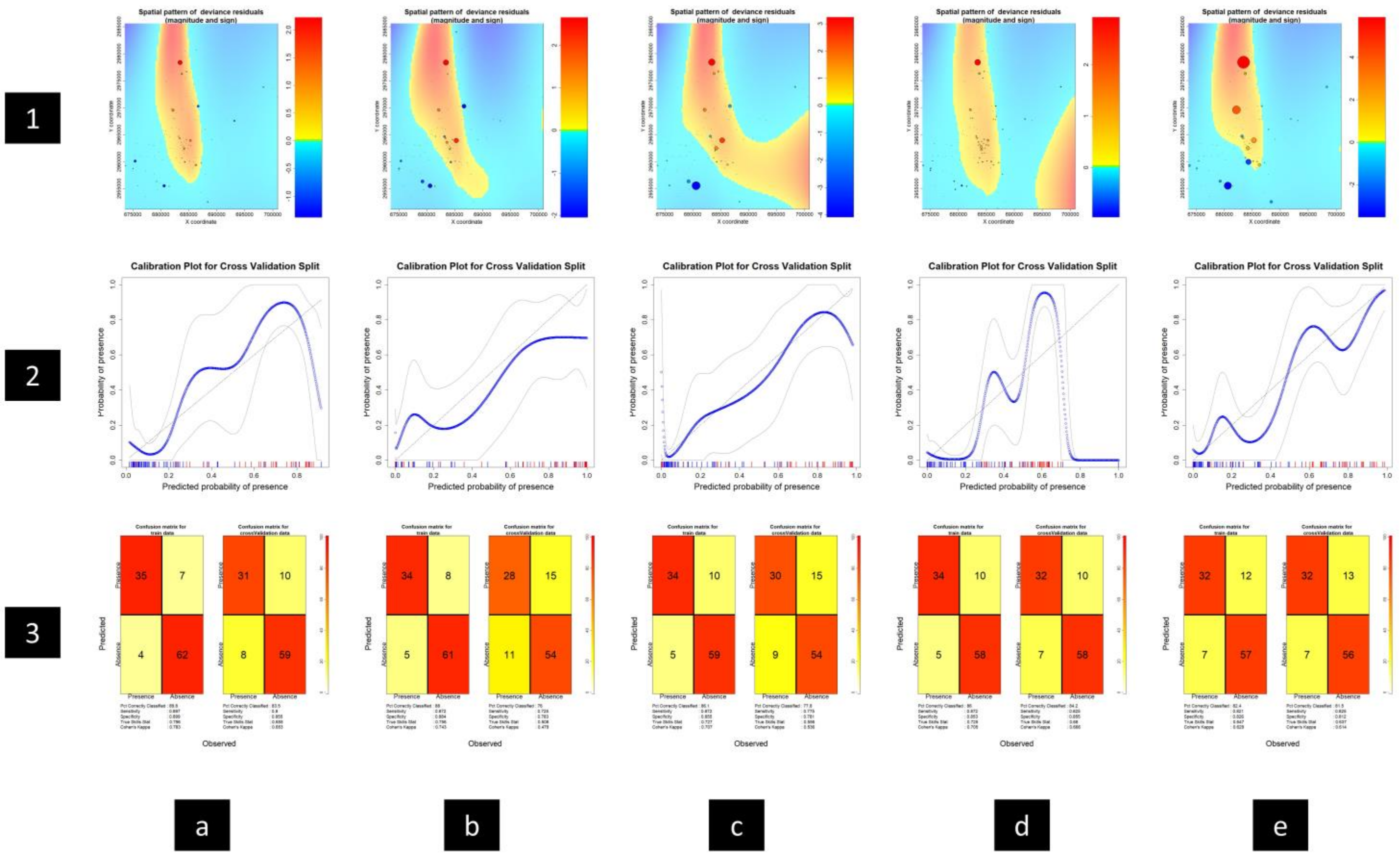

Supplementary Figure. S10. Evaluation Matrix performance across model runs. Brown- represents the correlation coefficient among the 5 different models. **Yellow-** represents the proportion of deviance explained; **Green-** represents the Proportion of correctly classified; **Blue-** represents Area under curve (AUC) and **Pink-** represents true skill statistics.

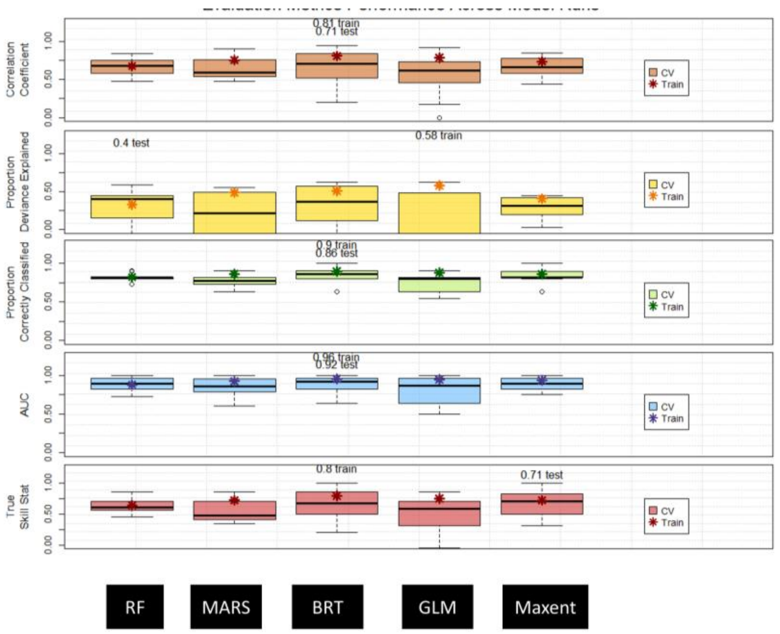

Supplementary Figure. S11. **Ensemble probability surfaces from 1998 to 2028.** Ensemble models of average habitat suitability from 5 different modelling algorithms including generalized linear model (GLM), multivariate adaptive regression spines (MARS), boosted regression tree (BRT), Random forest (RF) and Maximum entropy (MaxEnt) for Rhino in Gorumara National park along with its buffer forests including Chapramari and Bridge area. Starting from left represents the ensemble suitability for the year 1998 followed by 2008, 2018 and on extreme right hand, represents habitat suitability for future landscape 2028. All maps were generated using ArcGIS Ver. 10.6.

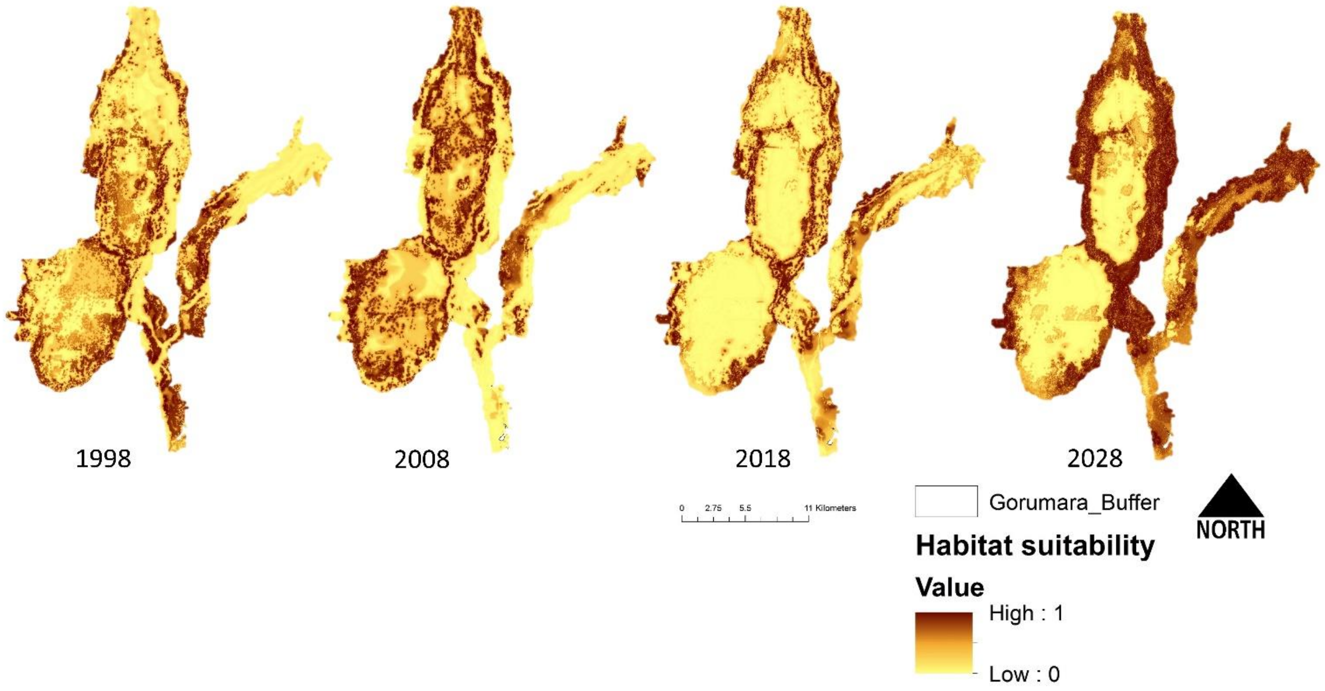

Supplementary Table S8. Change in most suitable area across multitemporal ensemble surfaces. Computed on the basis of maximum model agreement i.e. 5, from 1998 to 2008; 2008 to 2018 and fro, 2018 to 2028.

**1998**	**2008**	**Δ**	**1998%**	**2008%**	**Δ_%**
53.95 Km^2	60.59 Km^2	6.64 Km^2	16.976	19.066	2.090
**2008**	**2018**	**Δ**	**2008%**	**2018%**	**Δ_%**
60.59 Km^2	53.10 Km^2	-7.49 Km^2	19.066	16.708	-2.358
**2018**	**2028**	**Δ**	**2018%**	**2028%**	**Δ_%**
53.10 Km^2	97.15 Km2	44.05 Km^2	16.707	30.569	13.861

Supplementary Figure. S12. Simplified Methodological flow chart of the study. Where blue boxes represents the major section of the work design and arrow represents the direction of the work flow.

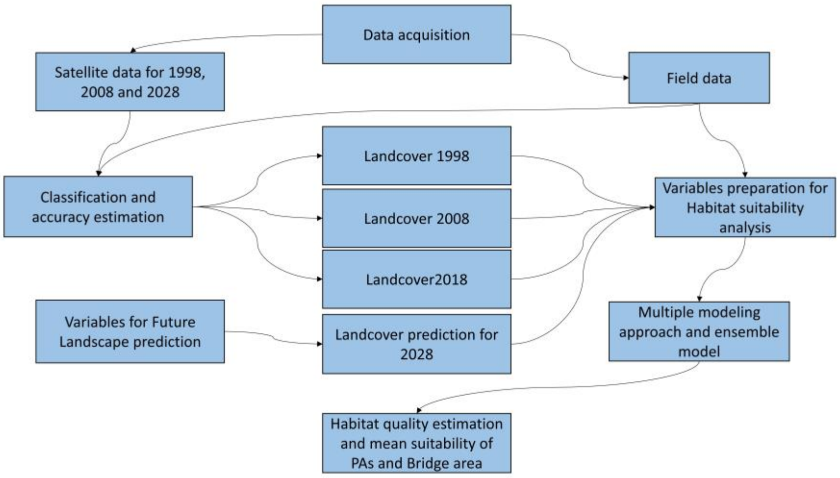

Supplementary Table S9. List of all eco-geographical variables for suitability modeling. Eco-geographical variables were categorised into two scales i.e. Land cover class-level variables and Landscape-level variables. The landcover class-level variables were generated by vectorization of the landcover classes followed by generating euclidian distance using ArcGIS 10.6 software. Whereas the landscape-level metrics were computed by using the moving window function of FRAGSTAT Ver. 4.2 software.

Sl. No.	**Variable**	**Code**	**Type**
**Landscape-level variables**			
**1.**	Number of Patches	NP	Continuous
**2.**	Patch Density	PD	Continuous
**3.**	Total Edge	TE	Continuous
**4.**	Edge Density	ED	Continuous
**5.**	Landscape Shape Index	LSI	Continuous
**6.**	Contagion	CONTAG	Continuous
**7.**	Percentage of Like Adjacencies	PLADJ	Continuous
**8.**	Interspersion & Juxtaposition Index	IJI	Continuous
**9.**	Patch Cohesion Index	COHESION	Continuous
**10.**	Landscape Division Index	DIVISION	Continuous
**11.**	Effective Mesh Size	MESH	Continuous
**12.**	Splitting Index	SPLIT	Continuous
**13.**	Patch Richness Density	PRD	Continuous
**14.**	Shannon's Diversity Index	SHDI	Continuous
**15.**	Patch Area Distribution (area-weighted mean)	area_am	Continuous

**16.**	Aggregation Index	AI	Continuous
**Landcover class-level variables**			
**17.**	Euclidian distance function from bare land landcover type	Bare_land	Continuous
**18.**	Euclidian distance function from grassland landcover type	Grassland	Continuous
**19.**	Euclidian distance function from river bank landcover type	River_bank	Continuous
**20.**	Euclidian distance function from shrubland landcover type	Shrubland	Continuous
**21.**	Euclidian distance function from water	Water	Continuous
**22.**	Euclidian distance function from woodland landcover type	Woodland	Continuous
